# Identification of a Sulfatase that Detoxifies Glucosinolates in the Phloem-Feeding Insect *Bemisia tabaci* and Prefers Indolic Glucosinolates

**DOI:** 10.3389/fpls.2021.671286

**Published:** 2021-06-04

**Authors:** Abinaya Manivannan, Bhawana Israni, Katrin Luck, Monika Götz, Elena Seibel, Michael L. A. E. Easson, Roy Kirsch, Michael Reichelt, Beate Stein, Stephan Winter, Jonathan Gershenzon, Daniel Giddings Vassão

**Affiliations:** ^1^Max Planck Institute for Chemical Ecology, Jena, Germany; ^2^Leibniz Institute DSMZ-German Collection of Microorganisms and Cell Cultures, Braunschweig, Germany

**Keywords:** whitefly, phloem-feeder, glucosinolates, sulfatase, pre-emptive detoxification, *Bemisia tabaci* MEAM1

## Abstract

Cruciferous plants in the order Brassicales defend themselves from herbivory using glucosinolates: sulfur-containing pro-toxic metabolites that are activated by hydrolysis to form compounds, such as isothiocyanates, which are toxic to insects and other organisms. Some herbivores are known to circumvent glucosinolate activation with glucosinolate sulfatases (GSSs), enzymes that convert glucosinolates into inactive desulfoglucosinolates. This strategy is a major glucosinolate detoxification pathway in a phloem-feeding insect, the silverleaf whitefly *Bemisia tabaci*, a serious agricultural pest of cruciferous vegetables. In this study, we identified and characterized an enzyme responsible for glucosinolate desulfation in the globally distributed *B. tabaci* species MEAM1. In *in vitro* assays, this sulfatase showed a clear preference for indolic glucosinolates compared with aliphatic glucosinolates, consistent with the greater representation of desulfated indolic glucosinolates in honeydew. *B. tabaci* might use this detoxification strategy specifically against indolic glucosinolates since plants may preferentially deploy indolic glucosinolates against phloem-feeding insects. *In vivo* silencing of the expression of the *B. tabaci* GSS gene via RNA interference led to lower levels of desulfoglucosinolates in honeydew. Our findings expand the knowledge on the biochemistry of glucosinolate detoxification in phloem-feeding insects and suggest how detoxification pathways might facilitate plant colonization in a generalist herbivore.

## Introduction

Plants rely on a complex arsenal of toxic chemicals to defend themselves against herbivores and pathogens. One successful defense strategy used to safely accumulate large concentrations of defensive compounds while preventing auto-toxicity is the production of two-component activated defenses, such as cyanogenic glucosides and glucosinolates (Halkier and Gershenzon, 2006; Mithöfer and Boland, 2012). These glucosylated pro-toxins are stable and only become toxic after enzymatic activation coincident with herbivore damage. Multi-component plant defenses like this, however, offer herbivores multiple targets for counter-adaptation, including mechanisms to prevent or redirect activation, or detoxify the activated poisons (Pentzold et al., 2014; Jeschke et al., 2016b). Piercing–sucking, phloem-feeding insects, such as aphids and whiteflies, are thought to lessen plant defensive responses by causing only minimal damage during feeding, stealthily penetrating plant tissues to feed on the sugar-rich phloem sap. Nevertheless, even piercing–sucking feeding leads to some activation of glucosinolates (Kim et al., 2008; Danner et al., 2018), so that detoxification strategies preventing activation of two-component defenses can be greatly advantageous to phloem-feeding insects. Here, we examine the biochemical basis of one such detoxification in the silverleaf whitefly *Bemisia tabaci* (Gennadius) (Hemiptera): the desulfation of glucosinolates to form derivatives that can no longer be activated.

**FIGURE 1:**
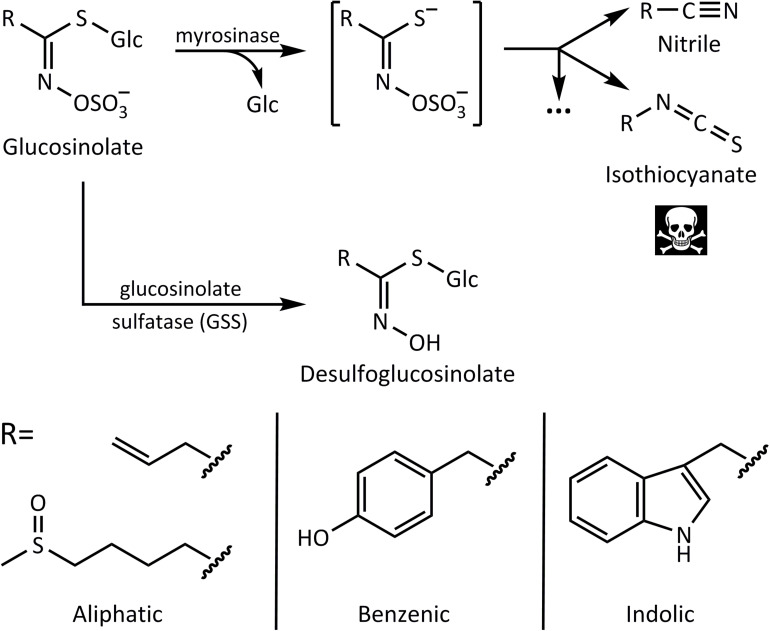
Glucosinolate activation by myrosinase leads to nitriles and toxic isothiocyanates, whereas desulfation by glucosinolate sulfatase (GSS) forms desulfoglucosinolates that cannot be activated. Below are examples of side chains of aliphatic, benzenic, and indolic glucosinolates derived from different amino acid precursors.

Glucosinolates are sulfur-containing defensive metabolites restricted to the plant order Brassicales. The family Brassicaceae of this order (mustard family) includes many economically important oilseed, vegetable, condiment, and fodder crops, such as rapeseed, cabbages, and mustards, as well as the model plant *Arabidopsis thaliana* (L.) Heynh. These plants have distinctive tastes and smells that are conferred in great part by hydrolysis products from glucosinolates, also called mustard oils (Halkier, 2016). The core chemical structure of glucosinolates (Figure 1) consists of a β-thioglucose residue, a sulfonated oxime moiety, and a variable amino acid-derived side chain group R (Halkier and Gershenzon, 2006). A recent review lists up to 137 natural glucosinolates described based on the variability at the R group (Blažević et al., 2020), with these being classifiable according to the precursor amino acid from which they are derived, such as aliphatic (e.g., from Met, Ala, and Val), benzenic (Phe and Tyr), and indolic (Trp).

In addition to glucosinolates, cruciferous plants produce thioglucosidase enzymes known as myrosinases that catalyze glucosinolate hydrolysis (Rask et al., 2000). Myrosinases and glucosinolates are physically segregated in the intact plant (Grob and Matile, 1979; Höglund et al., 1991). While the localization of each component is still a topic of study, myrosinases have been reported in protein-accumulating idioblasts called myrosin cells present in the phloem parenchyma. Glucosinolates, on the other hand, are enriched in sulfur-containing S-cells and would thus be separated from myrosinases at a cellular level (Koroleva et al., 2000). However, in other spatial separation models, glucosinolates are confined into the vacuoles, whereas myrosinases are present in the cytoplasm of the same cells, enabling compartmentalization at a subcellular level (Lüthy and Matile, 1984). Upon tissue disruption, such as during herbivory, this compartmentalization is compromised, and myrosinases hydrolyze the glucose moiety of the glucosinolate forming a mixture of toxic products (Figure 1). Isothiocyanates are considered the most toxic components of the mixture. This is attributed to their polarity and reactivity. The lipophilic nature of the isothiocyanate side chains allows them to easily cross cellular membranes, and the electrophilic nature of the –S=C=N group promotes reaction with nucleophilic targets (Kawakishi and Kaneko, 1987). The release of toxic isothiocyanates from glucosinolates is often referred to as the “mustard oil bomb.” By allowing the accumulation of large (millimolar) concentrations of glucosinolates that are only activated into more chemically reactive toxins upon herbivory, this two-component system serves as an important chemical defense against most herbivores (Hopkins et al., 2009; Jeschke et al., 2016a; Schweizer et al., 2017).

Several species of insect herbivores, however, have developed means to detoxify glucosinolates and successfully feed on crucifer plants with relative impunity (Jeschke et al., 2016b). For example, the isothiocyanate hydrolysis products can be converted to hydrophilic glutathione–isothiocyanate conjugates (Schramm et al., 2012; Jeschke et al., 2016a). This reaction, which takes place in many organisms including humans, can occur very efficiently in certain insects adapted to crucifers. On the other hand, the larvae of some crucifer-feeding *Pieris* species use a “nitrile specifying protein” for directing glucosinolate hydrolysis toward the formation of less toxic nitriles rather than isothiocyanates (Wittstock et al., 2004). Another enzymatic detoxification mechanism is the action of glucosinolate sulfatases (GSSs) in the insect gut. These enzymes are used by crucifer specialists, such as the diamondback moth larvae [*Plutella xylostella* (L.)] (Ratzka et al., 2002) and the cabbage stem flea beetle [*Psylliodes chrysocephala* (L.)] (Ahn et al., 2019), as well as the generalist herbivores desert locust [*Schistocerca gregaria* (Forskål)] (Falk and Gershenzon, 2007) and the silverleaf whitefly (*B. tabaci*) (Malka et al., 2016). GSSs cleave the sulfate group of glucosinolates to form desulfoglucosinolates (Figure 1), essentially reversing the last step in the biosynthesis of the glucosinolate core structure. As desulfoglucosinolates can no longer be used as substrates by myrosinases, no toxic downstream products are produced.

Among the insects that can desulfate glucosinolates is *B. tabaci*, a piercing-sucking, phloem-feeder that inhabits mainly tropical and subtropical regions. *B. tabaci* is a cryptic species complex, existing as a conglomerate of dozens of species (Perring, 2001) that are difficult to distinguish even with molecular markers (Boykin et al., 2012, 2018). The most invasive and geographically widespread species are MED (Mediterranean, formerly named Q) and MEAM1 (Middle East–Asia Minor 1, formerly B). *B. tabaci* species are altogether extreme generalists with a wide host plant range, colonizing more than 600 plant species including food and ornamental crops (Oliveira et al., 2001), and are, therefore, one of the most serious agricultural pests in the world. *B. tabaci* has been recently reported to desulfate glucosinolates with the resulting desulfoglucosinolate products excreted in honeydew (Malka et al., 2016). These transformation products were not observed in honeydew from other phloem feeders examined [the green peach aphid *Myzus persicae* (Sulzer) and the cabbage whitefly *Aleyrodes proletella* (L.) – unpublished data], suggesting that desulfation is not widespread in hemipteran insects. Indeed, among the insects currently known to detoxify glucosinolates by desulfation, *B. tabaci* is the only phloem-feeder. This specialized feeding mode minimizes mechanical damage during feeding, but still leads to detectable glucosinolate activation (Danner et al., 2018), especially of indolic glucosinolates (Kim et al., 2008). Pre-emptive deactivation of glucosinolates, such as via GSS activity, may therefore confer benefits to phloem-feeding insects as well.

In this study, we aimed to identify and characterize the enzyme(s) performing glucosinolate desulfation in *B. tabaci*. Several genes encoding potential GSSs were cloned and heterologously expressed, and the resulting proteins were tested for activity. However, only a single GSS was observed to efficiently desulfate glucosinolates in *B. tabaci*, and this enzyme had a strong preference for indolic glucosinolates. Silencing of *BtGSS* gene expression *in vivo* reduced the proportion of desulfoglucosinolates in the excreted honeydew relative to intact glucosinolates, confirming its role in glucosinolate metabolism in this insect.

## Materials and Methods

### Plants and Insects

*Bemisia tabaci* (MEAM1, formerly known as biotype B) was reared in the Leibniz Institute DSMZ (German Collection of Microorganisms and Cell Cultures), Braunschweig, Germany and in the Hebrew University of Jerusalem, Israel on eggplant [*Solanum melongena* (L.)] and Brussels sprouts [*Brassica oleracea* (L.)] plants. *A. thaliana* Col-0 was cultivated in a controlled-environment growth chamber under short day conditions (9.5:13.5 h, L:D, 140 μmol/m^2^/s photosynthetic photon flux density, 21°C, 50–60% relative humidity).

### Chemicals

Solvents used were HPLC grade and were obtained from VWR (Darmstadt, Germany). Glucosinolates were obtained from Sigma-Aldrich (Munich, Germany) and Phytoplan (Heidelberg, Germany). Arylsulfatase from *Helix pomatia* was obtained from Sigma-Aldrich and further processed as in Graser et al. (2000) and was used for preparation of desulfoglucosinolate standards by incubation of pure glucosinolates.

### *Bemisia tabaci* Sulfatase Candidate Selection and Their Tissue-Specific Expression Levels

The gene sequences of the putative *B. tabaci* MEAM1 GSSs were obtained from the Whitefly Genome Database (whiteflygenomics.org, downloaded January 26, 2017) and NCBI (downloaded July 11, 2018). *B. tabaci* arylsulfatase gene candidates were picked based on BLAST similarities to the sequence of the previously published *P. xylostella* GSS (PxGSS, Px018104^1^). The initial screening of *B. tabaci* sulfatase candidates included the estimation of ratios of gene expression between dissected *B. tabaci* gut tissues and whole insect bodies, using previously published data (Ye et al., 2014; Luan et al., 2015) SRR835757 and SRR1523522, previously also used for gene expression analysis in Jing et al. (2016). Expression levels in FPKM were estimated using DNAStar QSeq with the software parameters used in Vogel et al. (2014).

### Phylogenetic Analysis of GSSs and Arylsulfatase Candidates

A multiple alignment of the nucleotide sequences of the putative *B. tabaci* sulfatases and *P. xylostella GSS* was performed using the MUSCLE algorithm (Edgar, 2004) in the Geneious Prime software (default parameters, eight maximum iterations). The phylogenetic relationships among sequences were inferred using the neighbor-joining method and the Tamura-Nei genetic distance model, with the reliability of the tree branching tested with 1,000 bootstrap replicates, using the Geneious software.

### Primer Design, PCR Amplification, and Cloning of Candidate Genes

The gene sequences of the putative *B. tabaci* MEAM1 GSSs (obtained as described above) were used for the design of primers for the amplification of the respective open reading frames (ORFs). Primer design was carried out using the Geneious software (version 10.0.5; Biomatters Ltd., Auckland, New Zealand). Full-length ORFs were amplified using Phusion HF Polymerase (Thermo Scientific, Darmstadt, Germany) using *B. tabaci* cDNA as template for amplification. DNA fragments were then extracted from agarose gels using either QIAquick Gel Extraction Kit (Qiagen, Hilden, Germany) or the Zymoclean Gel DNA Recovery Kit (Zymo Research, Freiburg im Breisgau, Germany) as per the manufacturer’s instructions and cloned into the pIB/V5-His TOPO TA expression vector (Thermo Scientific). Final plasmids were transfected into Sf9 cells by lipid-mediated transfection using FuGENE Transfection Reagent (Promega, Mannheim, Germany) according to the manufacturer’s instructions for recombinant protein expression. A pIB/V5-His TOPO TA plasmid containing the corresponding full-length *PxGSS* from *P. xylostella* was also transfected and used as a positive expression and activity control. *Spodoptera frugiperda* Sf9 cells (Life Technologies, Darmstadt, Germany) were cultured in Sf-900 II serum-free medium (Life Technologies). Adherent cell cultures were maintained at 27°C and subcultured every 3–4 days. Collected cells and media were separated by gentle centrifugation at 500 × *g* for 5 min, with cell pellets then re-suspended and homogenized to generate crude cell-free protein extracts.

### Screening *Bemisia tabaci* Arylsulfatase Candidates for GSS Activity

To identify *B. tabaci* GSSs, *in vitro* enzyme assays were performed with crude protein extracts and media of cells heterologously expressing *B. tabaci* arylsulfatases. Reactions were performed in Tris buffer (100 mM, pH 7.5), to which 2–5 mM of glucosinolates being tested was added. The reactions were incubated at 25°C for 30 min and stopped using acetic acid (10% v/v). Assays were performed in duplicates for screening and triplicates for product quantification. Enzymatic assays with recombinant *P. xylostella* GSS were used as a positive control. The formation of desulfated glucosinolate products was quantified by high-performance liquid chromatography mass spectrometry (HPLC–MS/MS) using external calibration curves.

### Polyhistidine-Tagged Protein Purification

The polyhistidine tagged, heterologously produced *B. tabaci* GSS BtGSS was affinity-purified from the extracellular culture medium. First, the medium was collected and concentrated to 1 ml using centrifugal filter units (Amicon Ultra Centrifugal Filters; Merck, Darmstadt, Germany) with a molecular weight cut-off of 10,000 Da. Purification over Ni-NTA agarose resin (Qiagen, Hilden, Germany) followed the protocol of Eakteiman et al. (2018).

### Determination of *B. tabaci* GSS Optimal Temperature and pH

For determination of optimal reaction temperature, BtGSS was incubated with allyl glucosinolate (2 mM) in Tris buffer (100 mM, pH 7.5) for 10 min at 10, 15, 25, 35, 45, 55, 65, and 75°C. The enzyme reactions were stopped using acetic acid (10% v/v). Enzymatic assays were performed in duplicate. The desulfated allyl glucosinolate product formed was quantified by HPLC–MS/MS.

For determination of optimum pH, BtGSS was incubated with allyl glucosinolate (2 mM) in each of the following buffers: broad range: phosphate citrate (0.2 M phosphate, 0.1 M citrate; pH 3.0, 4.0, 5.0, 6.0, 7.0), Tris–HCl (0.1 M; pH 7.0, 8.0), and glycine–NaOH buffer (0.2 M; pH 8.0, 9.0); narrow range: sodium phosphate (0.1 M; pH 6.25, 6.50, 6.75, 7.00, 7.25, 7.50, 7.75, 8.00). Reactions were carried out for 10 min at 25°C and stopped using acetic acid (10% v/v). Assays were performed in duplicate. The desulfated allyl glucosinolate product formed was quantified by HPLC–MS/MS.

### Quantitative Real-Time PCR Analysis of Basal *BtGSS* Expression

To check for the expression levels of *BtGSS*, quantitative real-time PCR (qPCR) analyses were performed using cDNA generated from four biological replicates of *B. tabaci* reared on eggplant (non-glucosinolate-containing diet) and Brussels sprouts (glucosinolate-containing diet). Ribosomal protein L-13 (*Bta04282*) was used as a reference gene. Primers were designed with an optimal melting temperature of 60°C. The list of primers is available in Supplementary Table 1. Each reaction was done in triplicate. Expression levels of *BtGSS* were relatively quantified using the 2^–ΔΔCT^ method (Pfaffl, 2001) and are presented as means ± standard errors.

### Silencing of *BtGSS* Expression *in vivo* and Resulting Metabolic Changes

Silencing experiments were carried out using artificial diets containing sucrose, 4msob glucosinolate, and two double-stranded RNases (dsRNAs), one targeting dsRNase2 and the other BtGSS, or a scrambled BtGSS-derived sequence used as a negative control (synthesis and cloning into pUC57 backbone by GenScript, Leiden, Netherlands). Initial amplification was carried out from *B. tabaci* cDNA (or pUC57 plasmid for the scrambled sequence) as template, using gene-specific primers containing the T7 polymerase site (Supplementary Table 2). The PCR products were used as templates for *in vitro* transcription reactions using the MEGAscript kit (Thermo Fisher Scientific). The products obtained were quantified using Nanodrop (Thermo Fisher Scientific), and the integrity of the dsRNA was confirmed on a 1.2% denaturing agarose gel. Feeding experiments were carried out in glass vials, with 50–100 whiteflies per vial. Aqueous artificial diets were enclosed between two thin layers of stretched parafilm and contained 0.5 μg/μl BtGSS (or scrambled BtGSS) dsRNA, 0.5 μg/μl dsRNase2 dsRNA, 0.29 M sucrose, and 5 mM 4msob glucosinolate. Silencing was carried out over a period of 48 h. At the end of the feeding period, whiteflies and honeydew were collected for gene expression and metabolite analysis, respectively. Whiteflies were homogenized in 500 μl TRIzol reagent (Thermo Fisher Scientific) for total RNA isolation, and cDNA synthesis was carried out with 0.5 μg total RNA using SuperScript IV Reverse Transcriptase (Thermo Fisher Scientific) according to the manufacturer’s protocol. Honeydew samples were processed by washing the glass vials twice with 500 μl methanol, concentrating under nitrogen flow and re-suspending in 200 μl water. Honeydew samples were used for quantification of 4msob desulfoglucosinolate and non-metabolized 4msob glucosinolate by HPLC–MS/MS.

### Glucosinolate Extraction From *Arabidopsis thaliana*

Leaves from *A. thaliana* Col-0 rosette stage plants were harvested, flash frozen using liquid N_2_, and freeze-dried (Alpha 1-4 LDplus freeze dryer; Martin Christ, Osterode am Harz, Germany). Intact glucosinolates were extracted with 10 ml 80% (v/v) methanol:water per g dry weight (DW) on ice under continuous shaking for 5 min, followed by the addition of metal beads (3 mm) and vigorous shaking for 10 min in a paint shaker. The supernatant collected after centrifugation (4,000 × *g* at 4°C for 15 min) was then processed through centrifugal filter units (Amicon Ultra Centrifugal Filters) with a molecular weight cut-off of 10,000 Da to separate glucosinolates from the plant myrosinase. The flow-through was then treated on a rotatory evaporator for removal of the solvent and was resuspended in water.

### Tests of *Bemisia tabaci* Sulfatase Substrate Preference With *Arabidopsis* Glucosinolates

To test the substrate preference of the *B. tabaci* GSS, enzyme assays were performed with the glucosinolate extract obtained from *A. thaliana* Col-0 leaves. Purified BtGSS was added to Tris buffer (100 mM, pH 7.5) and incubated with glucosinolate extract at 25°C. Aliquots were taken at different time points (0–300 min) and mixed with acetic acid (10% v/v). Enzymatic assays were performed in duplicate, and no-enzyme reactions served as a control for non-enzymatic glucosinolate degradation. The intact glucosinolates remaining in each reaction sample were quantified by HPLC–MS/MS.

### Quantitative Tests of BtGSS Substrate Preference With Selected Pure Glucosinolates

To test the substrate preference of the *B. tabaci* GSS, enzyme assays were performed with selected pure glucosinolates: 4msob-, i3m-, allyl-, and *p*OHBn-glucosinolates. Purified BtGSS enzyme (1 μg) was added to Tris buffer (0.1 M, pH 7.5, 100 μl total volume) and was incubated with 2 mM glucosinolate extract at 25°C. Reactions proceeded for 10 min and were then stopped with acetic acid (10% v/v). Enzymatic assays were performed in triplicate, and no-enzyme reactions served as a control for non-enzymatic glucosinolate degradation. The desulfoglucosinolates formed from 4msob and i3m glucosinolates were quantified relative to external calibration curves of the respective products by HPLC–MS/MS, as described below.

### High-Performance Liquid Chromatography Mass Spectrometry

Analyses of desulfated and intact glucosinolates in honeydew and enzyme assays were performed on an Agilent Technologies (Santa Clara, CA, United States) 1200 Series HPLC using a Nucleodur Sphinx RP column (250 × 4.6 mm × 5 μm; Macherey-Nagel, Düren, Germany) coupled to an API 3200 triple-quadrupole mass spectrometer (Applied Biosystems, Darmstadt, Germany). Formic acid (0.2%) in water and acetonitrile were employed as mobile phases A and B, respectively. The flow rate was 1.1 ml/min. The elution profile was as follows: 0–2.5 min, 1.5% B; 2.5–5 min, 1.5–10% B; 5–12.5 min, 10–40% B; 12.5–17.5 min, 40–70% B; 17.6–20 min, 100% B; and 20.1–24 min, 1.5% B. The ion spray voltage was maintained at 4,500 eV. The turbo gas temperature was set to 700°C. Nebulizing gas was set at 70 psi, curtain gas at 20 psi, heating gas at 60 psi, and collision gas at 10 psi. Multiple reaction monitoring (MRM, Supplementary Tables 3, 4) was used to monitor parent ion to fragment ion conversion (Malka et al., 2016). Analyst 1.5 software (Applied Biosystems) was used for data acquisition and processing.

Analyses of desulfated and intact glucosinolates during RNAi silencing experiments were performed on an identical HPLC setup as those above, but coupled to an API 6500 triple-quadrupole mass spectrometer (Applied Biosystems). Formic acid (0.05%) in water and acetonitrile were employed as mobile phases A and B, respectively. The elution profile was identical to the abovementioned but with a flow rate of 1.0 ml/min. Ion spray voltages were maintained at 4,500 eV (positive mode for desulfoglucosinolates) and -4,500 eV (negative mode for intact glucosinolates). The turbo gas temperature was set to 650°C. Curtain gas was set at 40 psi, collision gas at medium, ion source gas 1 at 60 psi, and ion source gas 2 at 60 psi. MRM was used to monitor parent ion to fragment ion conversion (Supplementary Table 5). Analyst 1.6.3 software (Applied Biosystems) was used for data acquisition and processing.

## Results

### Identification of Putative *Bemisia tabaci* GSS

**FIGURE 2:**
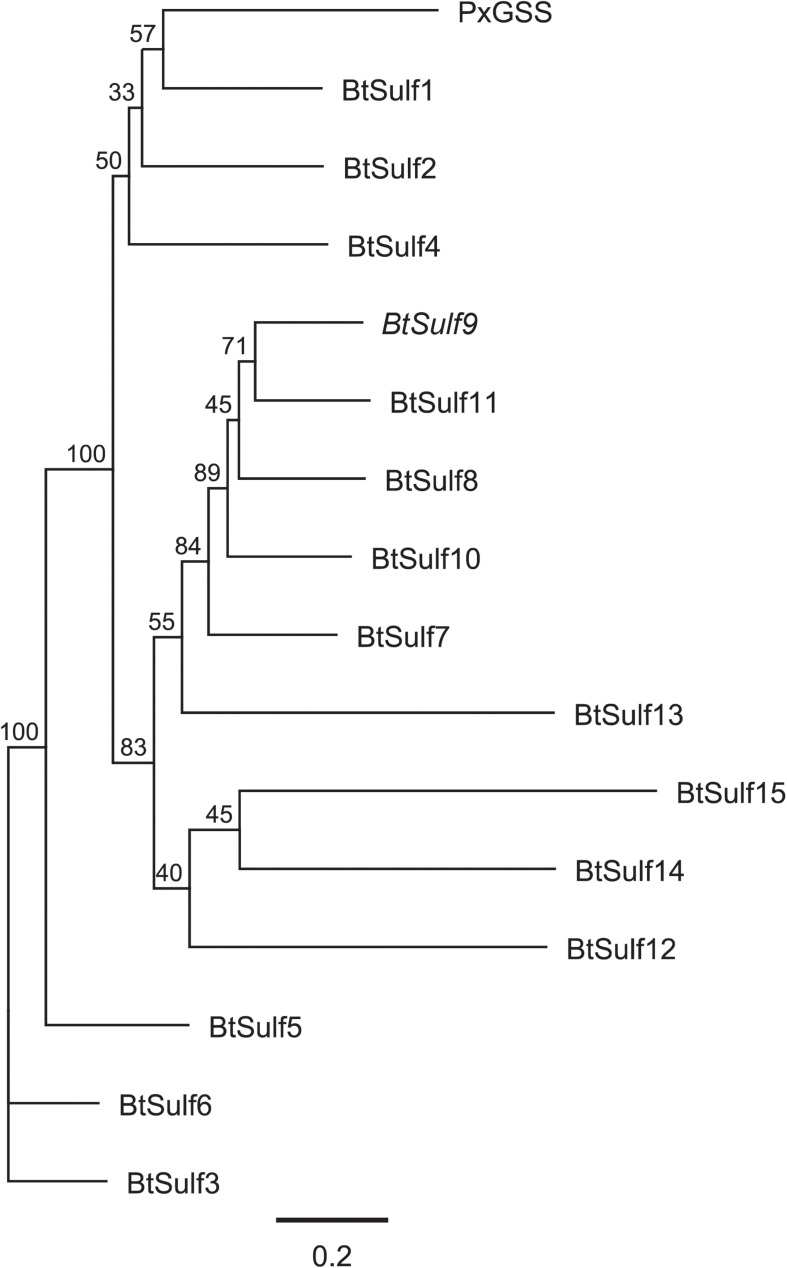
Phylogenetic relationships of predicted *Bemisia tabaci* sulfatases (BtSulf), including BtSulf9 (BtGSS, italicized) and *P. xylostella* GSS (PxGSS). Gene names are as described in Table 1, and bootstrap values (%) are shown next to each node.

The protein sequence of the previously identified *P. xylostella* GSS (Ratzka et al., 2002) was used as a query to identify *B. tabaci* sulfatases (BtSulf) in the published *B. tabaci* MEAM1 draft genome (Chen et al., 2016) that might serve as GSSs. Candidates were sought that had high similarity to PxGSS and a high expression level of the encoding genes in *B. tabaci* guts relative to the whole insect based on publicly available expression data (Table 1 and Figure 2). Most candidates had been previously annotated as arylsulfatases. Selected sequences were cloned from *B. tabaci* (species MEAM1) whole-insect cDNA and heterologously expressed in Sf9 cells, and the produced protein was subsequently screened for GSS activity.

**TABLE 1:** Predicted *Bemisia tabaci* sulfatase (“BtSulf”) candidates, ordered based on sequence similarity scores (e-values) to *Plutella xylostella* GSS (PxGSS).

				MEAM			MED	
					
NCBI entry	Bta entry	Name	e-value (vs. PxGSS)	MEAM gut (FPKM)	MEAM adults (FPKM)	Expression ratios, gut vs. whole adults	MED gut (FPKM)	MED adults (FPKM)
XM 019054398	Bta06756	BtSulf1	2,60E-122	0	216	0	0	138
XM 019062599	Bta04898	BtSulf2	4,21E-112	0	81	0	0	20
XM 019061487	Bta03550	BtSulf3	1,18E-107	0	70	0	3	488
XM 019062598	Bta04899	BtSulf4	2,09E-107	0	248	0	1	275
XM 019051255	Bta02222	BtSulf5	1,50E-106	460	675	1,5 lower	370	945
XM 019052592	Bta04774	BtSulf6	1,11E-101	16	277	17,3 lower	5	103
XM 019059032	Bta14665	BtSulf7	5,36E-100	48	272	5,7 lower	81	201
XM 019058912	Bta14669	BtSulf8	1,02E-97	0	20	0	0	93
*XM 019059016*	*Bta14666*	*BtSulf9*	*4,87E-96*	*8526*	*1203*	*7,1 higher*	*7296*	*135*
XM 019049969	Bta01141	BtSulf10	2,88E-83	10	39	3,9 lower	96	62
XM 019059017	Bta14667	BtSulf11	1,76E-65	10	0	higher	2	0
XM 019042567	Bta08750	BtSulf12	5,73E-21	2627	9434	3,6 lower	1587	9732
XM 019054362	Bta06730	BtSulf13	7,94E-17	18	468	26,0 lower	12	746
XM 019049823	Bta01054	BtSulf14	3,13E-13	40	207	5,2 lower	15	55
XM 019053003	Bta05280	BtSulf15	3,59E-08	51	300	5,9 lower	38	360

*Genes are denoted according to their accession numbers in NCBI and the whitefly genome databases. Estimated expression levels (FPKM) for these genes in gut tissues and whole insect bodies were calculated from publicly available data for B. tabaci MEAM1 insects and, as a reference, also for B. tabaci MED insects.*

### *Bemisia tabaci BtSulf9* Encodes a GSS Enzyme (BtGSS)

**FIGURE 3:**
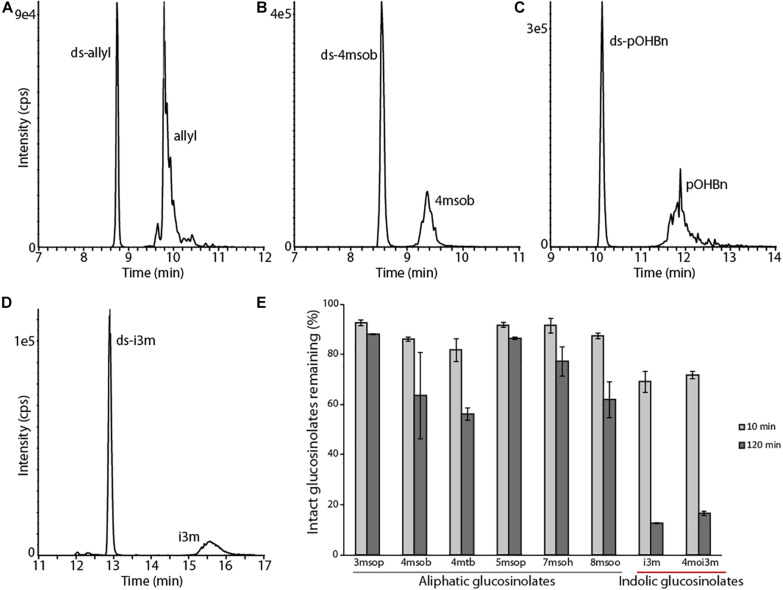
BtGSS has activity with a variety of glucosinolates. **(A–D)** LC–MS extracted MRM chromatograms of desulfated glucosinolates produced *in vitro* by BtGSS. The early eluting peaks in each chromatogram indicate genuine desulfoglucosinolates formed during the enzyme assay, whereas the later eluting peaks show desulfoglucosinolates formed from intact glucosinolates via in-source fragmentation during MS analysis and so actually indicate the presence of intact sulfated glucosinolates used as substrates. **(E)** The time-dependent degradation of a mixture of intact glucosinolates from *Arabidopsis thaliana* Col-0, with indolic glucosinolates being depleted more quickly than aliphatic ones (amount of each glucosinolate present at 0 min was taken as 100%, *N* = 2 independent reactions). “ds-” = desulfated glucosinolates; 3msop = 3-methylsulfinylpropyl; 4msob = 4-methylsulfinylbutyl; 4mtb = 4-methylthiobutyl; 5msop = 5-methylsulfinylpentyl; 7msoh = 7-methylsulfinylheptyl; 8msoo = 8-methylsulfinyloctyl; i3m = indol-3-ylmethyl; 4moi3m = 4-methoxyindol-3-ylmethyl.

Previous reports showed that PxGSS produced in *Escherichia coli* had no sulfatase activity (Ratzka et al., 2002), suggesting that those cells lacked the post-translational modification machinery necessary to generate an active GSS. Hence, insect cells (*S. frugiperda* Sf9 cells) were used here for expressing the *B. tabaci* GSS candidates. The full-length ORFs of selected *B. tabaci* sulfatases (BtSulf1, 3, 5–9, and 11) were successfully expressed to produce V5-/His-tagged proteins. The resulting *B. tabaci* enzymes were screened for their activity toward pure glucosinolates: allyl-(sinigrin), *p*-hydroxybenzyl-(*p*OHBn, sinalbin), and 4-methylsulfinylbutyl-(4-msob, glucoraphanin) glucosinolates. Heterologously produced PxGSS and commercial *H. pomatia* sulfatase were used as positive controls for GSS activity. The resulting desulfoglucosinolate products were detected by HPLC–MS/MS. Intact glucosinolates also gave rise to MS signals corresponding to desulfated glucosinolates, due to in-source fragmentation (spontaneous sulfate loss) in the mass spectrometer. However, these artifactual desulfated glucosinolates were readily distinguished from genuine desulfoglucosinolates existing before analysis due to different retention times. These analyses revealed that BtSulf9 had GSS activity toward all tested glucosinolates (Figure 3 and Supplementary Figure 1) and this was henceforth named BtGSS. Both BtGSS and PxGSS were secreted by the Sf9 cells into the extracellular culture medium. BtGSS purified by metal affinity chromatography had an optimum temperature around 50–55°C and an optimum pH between 7.5 and 7.8. None of the other produced sulfatases tested displayed detectable GSS activity *in vitro*, including BtSulf1 (the closest homolog to PxGSS) and those in the same phylogenetic branch as BtGSS (BtSulf7, 8, 11).

### *BtGSS* Expression Is Independent of Glucosinolate Ingestion

To compare the relative expression levels of *BtGSS* in *B. tabaci* reared on non-glucosinolate diet (eggplant) and those reared on a glucosinolate-rich diet (Brussels sprouts), we analyzed the expression of *BtGSS* by qPCR in *B. tabaci* MEAM1 adult insects grown on both plants. *BtGSS* was constitutively and highly expressed in insects grown on both plant species tested (Supplementary Figure 2).

### Silencing of BtGSS Supports Its Role as the Major GSS in *Bemisia tabaci*

**FIGURE 4:**
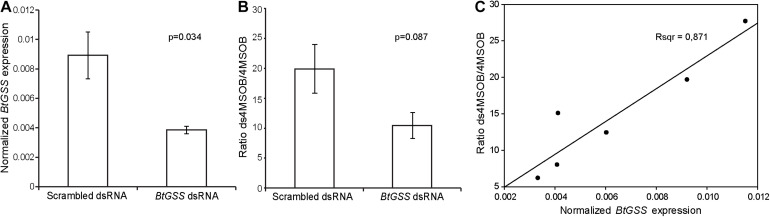
*BtGSS* silencing *in vivo*. **(A)** Dietary administration of dsRNA (*N* = 3 per treatment; statistical differences determined via *t*-tests) targeting *BtGSS* led to diminished expression of this gene (relative to *rpl-13*) compared with a treatment using a scrambled dsRNA control. **(B)** Silencing reduced the proportion of desulfo-4msob glucosinolate relative to intact glucosinolate present in whitefly honeydew. **(C)** *BtGSS* expression was positively correlated to the proportions of desulfo-4msob glucosinolate:intact glucosinolate in honeydew.

To examine whether BtGSS acts to desulfate glucosinolates *in vivo*, we manipulated the expression of its encoding gene *BtGSS* using an RNAi approach. For this purpose, dsRNA targeting BtGSS was synthesized using *in vitro* transcription and fed to *B. tabaci* adults in artificial diets. Since non-specific dsRNases in *B. tabaci* could cause dsRNA degradation in the whiteflies, artificial diets also contained dsRNA targeting dsRNase2, which had been previously shown to enhance the silencing of genes of interest (Luo et al., 2017). This strategy led to a decrease of 43% in the expression of *BtGSS* in *B. tabaci* compared with a scrambled dsRNA control (Figure 4A). After dsRNA treatment, the proportion of 4msob-desulfoglucosinolate relative to non-metabolized intact 4msob-glucosinolate in honeydew samples was 48% lower in *BtGSS*-silenced adults than in scrambled dsRNA-fed control insects (Figure 4B). Both the extent of *BtGSS* gene silencing and the differences in desulfoglucosinolate formation indicated a high variability among experimental groups. However, these two factors were linearly correlated, with higher *BtGSS* expression corresponding to higher proportions of desulfoglucosinolate products in honeydew (Figure 4C).

### BtGSS Shows Preference Toward Indolic Glucosinolates

To compare BtGSS activity toward glucosinolates with different side chains as substrates, the enzyme was incubated for different periods with pure glucosinolates and with a glucosinolate extract obtained from *A. thaliana* Col-0 leaves, which contained both aliphatic and indolic glucosinolates (Brown et al., 2003). BtGSS desulfated all glucosinolates offered, both as pure compounds and within the *A. thaliana* extract (Figures 3A–D and Supplementary Figure 1A–H). However, indolic glucosinolates were degraded much more rapidly than aliphatic glucosinolates (Figure 3E). All glucosinolates were stable under non-enzymatic control conditions (Supplementary Figure 3). This confirmed that BtGSS acts on different glucosinolate classes and suggested additionally that it might have distinct substrate affinities/catalytic efficiencies toward them. We confirmed this using two selected pure glucosinolates for *in vitro* incubations with BtGSS, 4msob- and i3m-glucosinolates. BtGSS desulfated i3m-glucosinolate, the most abundant indolyl glucosinolate in *A. thaliana* Col-0, with a catalytic efficiency of 466 nmol/mg BtGSS/min. This was 2.93 times more efficient than the conversion of 4msob-glucosinolate (159 nmol/mg BtGSS/min), its most abundant aliphatic counterpart in *A. thaliana* and several *Brassica* crops.

## Discussion

Although cruciferous plants possess a sophisticated glucosinolate–myrosinase defense system to thwart their herbivores, several insects possess mechanisms to detoxify these plant defensive metabolites. One such strategy is to employ a GSS enzymatic system to convert glucosinolates to their corresponding desulfated forms, and thus prevent them from being broken down to toxic downstream products by myrosinases (Ratzka et al., 2002; Falk and Gershenzon, 2007). Interestingly, while all previously described GSS activities had come from leaf-chewing herbivores, this detoxification mechanism was recently reported from the phloem-feeding herbivore *B. tabaci* (Malka et al., 2016). It was once believed that phloem-feeding insects, by delicate piercing of plant tissues and stealthy sucking of the plant sap, do not strongly activate the glucosinolate–myrosinase system (Walling, 2008; Pentzold et al., 2014). However, more recent studies showed that phloem feeding indeed leads to glucosinolate activation and production of isothiocyanates (Walling, 2008; Danner et al., 2018). These recent results clarify the advantage of efficiently and pre-emptively detoxifying glucosinolates in piercing–sucking insects, such as whiteflies. As the *B. tabaci* species complex is a major agricultural pest, it was of interest to identify the GSS(s) performing glucosinolate detoxification in this insect.

We successfully identified one GSS (BtGSS) in *B. tabaci* MEAM1 that actively desulfated different glucosinolates *in vitro*. The heterologously produced enzyme displayed optimum GSS activity at pH 7.5–7.8 and was secreted into the extracellular culture medium by Sf9 cells, in agreement with the presence of a native signal peptide guiding BtGSS for secretion. Additionally, available transcriptomic data suggested an elevated expression of this enzyme in the *B. tabaci* gut tissues relative to the rest of the body in both MEAM1 and MED *B. tabaci* species (formerly named “B” and “Q” biotypes, respectively). These data together suggest that this enzyme is secreted into the *B. tabaci* gut lumen, where it can efficiently detoxify potentially harmful glucosinolates after ingestion. This *modus operandi* mimics that of the *P. xylostella* GSS, which is secreted from gut epithelial cells and is present in high amounts in the larval gut lumen (Ratzka et al., 2002). Nevertheless, the *B. tabaci* BtGSS we identified is not the sulfatase with closest homology to the *P. xylostella* GSS in the whitefly. The sulfatase designated BtSulf1 is phylogenetically closest to PxGSS but did not have GSS activity *in vitro*. These data suggest that GSS activity evolved independently in whiteflies and lepidopterans from sulfatases performing different innate functions in these distant insect lineages. In fact, BtGSS was the only sulfatase for which GSS activity was detected among all BtSulf candidates tested in this study. These included BtSulf 7, 8, and 11, whose sequences were most closely related to BtGSS, but did not show GSS activity *in vitro*. Additionally, RNAi-mediated silencing of BtGSS in artificial diet-fed whiteflies led to a decrease in the proportion of desulfoglucosinolates in honeydew relative to unmetabolized intact glucosinolates. This suggests that BtGSS is indeed the major sulfatase responsible for glucosinolate desulfation in *B. tabaci* MEAM1.

We found that BtGSS exhibits a clear preference *in vitro* toward indolic glucosinolates rather than their aliphatic counterparts in *A. thaliana* Col-0. This result is in accordance with the higher proportion of indolic glucosinolates found to be desulfated relative to their aliphatic counterparts in honeydew of *B. tabaci* feeding on Brussels sprouts and *A. thaliana* (Malka et al., 2016). Thus, *B. tabaci* might employ its GSS detoxification machinery to specifically combat the induction and activation of indolic glucosinolates that takes place during phloem sap-feeding (Kim et al., 2008). This substrate preference also complements well another preemptive glucosinolate detoxification mechanism in *B. tabaci*, the formation of glucosylated glucosinolates. Analysis of honeydew from *B. tabaci* feeding on *A. thaliana* showed that glucosylation was found to occur mostly to aliphatic glucosinolates and few glucosylated indolic glucosinolate derivatives were detected (Malka et al., 2020).

The existence of efficient pre-emptive detoxification pathways in *B. tabaci* can help explain the apparent lack of preference of MEAM1 and MED whiteflies for plants with lower vs. higher glucosinolate contents (Yang et al., 2020); that is, as these *B. tabaci* species efficiently deactivate glucosinolates, they can feed on plants producing natural concentrations of glucosinolates with impunity. However, when glucosinolate production was increased above natural levels in transgenic lines, *B. tabaci* development and performance was negatively affected (Elbaz et al., 2012; Markovich et al., 2013). Moreover, *myb51*, a positive regulator of indolic glucosinolate production, is up-regulated in *A. thaliana* after infestations by the phloem-feeding insects *B. tabaci*, *M. persicae*, and *Brevicoryne brassicae* (Foyer et al., 2015). Taken together, these findings suggest that plants specifically induce the production of indolic glucosinolates as a response against phloem-feeding insects. These glucosinolates could be activated by one of the recently characterized non-canonical glucosinolate-β-thioglucosidases, such as PEN2 or PYK10 (Bednarek et al., 2009; Clay et al., 2009; Nakano et al., 2017), which may be more specific to indolic glucosinolates. Plants can also convert indol-3-ylmethyl glucosinolate into more bioactive methoxylated forms as a defensive response (Kim and Jander, 2007; Wiesner et al., 2013; Pfalz et al., 2016). The enzymatic breakdown products of indolic glucosinolates are said to have strong antifeedant and toxic effects on crucifer-consuming insects (Kim and Jander, 2007; Agerbirk et al., 2008; Kim et al., 2008).

Quantitative real-time PCR analysis showed that the gene encoding the identified *B. tabaci* GSS (*BtGSS*) is highly and constitutively expressed irrespective of whether the insect consumed glucosinolates or not. This suggests that this GSS could have other function(s) for the insect, perhaps serving as a more general arylsulfatase. While the *in vivo* functions of insect (aryl)sulfatases are still not well-defined, one of their proposed roles is during the molting process, for example, of southern armyworm larvae (Yang et al., 1973). It is known that sulfate conjugates of ecdysone, the steroidal insect molting hormone, are less bioactive than non-sulfated analogs and arylsulfatase titers increase upon molting (King, 1972; Karlson and Koolman, 1973; Yang et al., 1973). Arylsulfatases could, therefore, be involved in converting ecdysone to its active form. However, ecdysteroid localization is typically limited to ecdysteroidogenic glands and hemolymph (Gilbert et al., 2002). According to previous studies of the *P. xylostella* GSS and *S. gregaria* GSS, and in accordance with what we observed for *B. tabaci*, GSS is secreted into the gut lumen (Ratzka et al., 2002; Falk and Gershenzon, 2007). Therefore, it seems unlikely that BtGSS is involved in ecdysone metabolism, and its other potential roles *in vivo* remain unknown.

In this work, we have characterized the BtGSS gene expression levels and protein activities in *B. tabaci* of the MEAM1 species. Desulfoglucosinolates had, however, been previously reported in *B. tabaci* MED whiteflies (Malka et al., 2016), another aggressive, generalist *B. tabaci* species. Whether MED and other *B. tabaci* species might have different GSS activity levels, and how this might affect their establishment on crucifers, is not yet known. Nevertheless, it seems clear that *B. tabaci* uses several different biochemical strategies to deal with glucosinolates. Besides the serial glucosylation of glucosinolates (Malka et al., 2020), a glutathione-*S*-transferase enzyme has also been reported in *B. tabaci* (Eakteiman et al., 2018) that can metabolize the hydrolysis products of both major aliphatic and indolic glucosinolates present in *A. thaliana*. This latter activity might help to mitigate the potential negative effects caused by the small amounts of glucosinolates successfully activated during and after ingestion by neutralizing the toxic hydrolysis products. Whether other strategies might also be employed by this whitefly, as in another generalist phloem-feeding insect, *M. persicae* (Kim and Jander, 2007; Kim et al., 2008), remains to be determined.

## Conclusion

The glucosinolate–myrosinase system is a sophisticated two-component chemical defense strategy present in plants of the order Brassicales. The constituents of this binary defense system are kept spatially segregated, but during herbivory, myrosinases catalyze the hydrolysis of glucosinolates forming toxic breakdown products. In contrast to leaf-chewing insects, which cause extensive damage during herbivory and activate the glucosinolate–myrosinase system, it was long assumed that phloem-feeding insects did not cause sufficient damage for activation. However, glucosinolate hydrolysis products have been detected during attack by aphids; and these hydrolysis products negatively affect phloem-feeding insects. Moreover, plants induce the production of glucosinolates (especially indolic glucosinolates) upon infestation by phloem feeders. Therefore, the strategies used by phloem-feeding insects that feed on glucosinolate-containing plants to circumvent these defensive metabolites deserve further attention.

The whitefly *B. tabaci* is one such crucifer-colonizing, phloem-feeding insect, which has been reported to efficiently detoxify glucosinolates by conversion to their corresponding desulfated forms. This species complex is a group of serious agricultural pests that causes extensive damage to crops including cruciferous vegetables, and also vectors many plant pathogenic viruses. Here, we have successfully identified and characterized a *B. tabaci* MEAM1 sulfatase that is responsible for this metabolic reaction. This enzyme exhibits a strong preference *in vitro* toward indolic glucosinolates in comparison with aliphatic glucosinolates, implying that *B. tabaci* employs this GSS detoxification mechanism to cope with the negative effects of indolic glucosinolate breakdown. This strategy seems to complement well the other glucosinolate detoxification pathways present in *B. tabaci* (glucosylation and glutathione conjugation) and helps explain why this insect can feed widely on cruciferous plants. However, the relative importance of the individual pathways, their costs, and how each contributes to the success of *B. tabaci* still remains to be determined.

## Data Availability Statement

The datasets presented in this study can be found in online repositories. The names of the repository/repositories and accession number(s) can be found in the article/Supplementary Material.

## Author Contributions

DV designed this study with input from AM, SW, and JG. AM, KL, BI, MG, ES, MLAEE, and BS performed the data collection and analysis. RK and MR provided additional materials, expertise, and technical help. SW, JG, and DV supervised the study. AM and DV drafted the manuscript with input from all coauthors. All authors contributed to the article and approved the submitted version.

## Conflict of Interest

The authors declare that the research was conducted in the absence of any commercial or financial relationships that could be construed as a potential conflict of interest.

## Footnotes

1 http://iae.fafu.edu.cn/DBM/genePage.php?id=Px018104, downloaded on 01/15/2017, and http://www.insect-genome.com/search/gene_detail1.php?id=790752, downloaded on 05/18/2021
