# Supplementary material for: Identification of a Sulfatase that Detoxifies Glucosinolates in the Phloem-Feeding Insect Bemisia tabaci and Prefers Indolic Glucosinolates
Source: Front Plant Sci. 2021 Jun 4;12:671286. doi: 10.3389/fpls.2021.671286 (PMC8212129; doi:10.3389/fpls.2021.671286)
Supplement: Supplementary file 1 [file Data_Sheet_1.PDF]

Supplementary materials

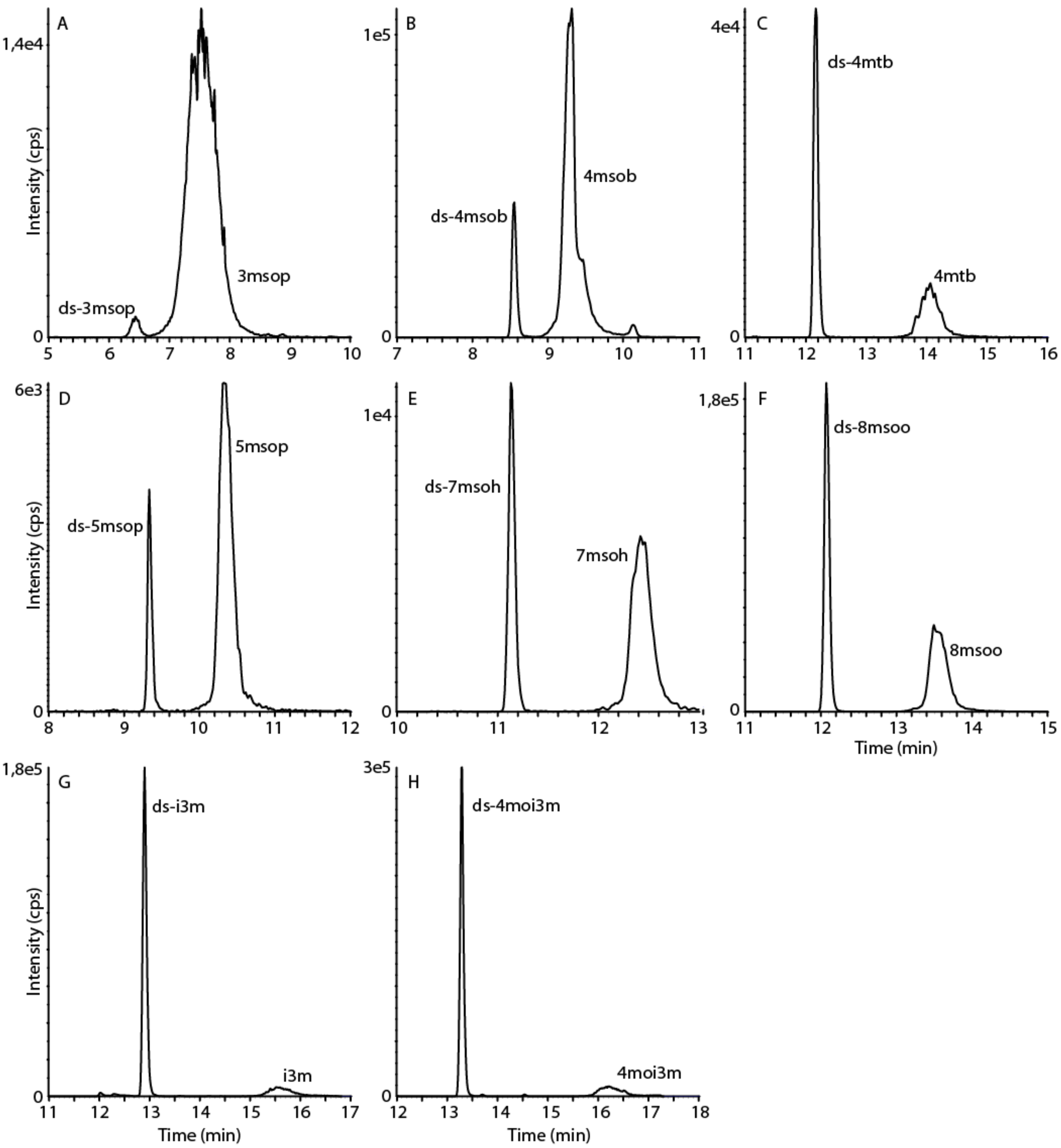

Figure Suppl. 1: BtGSS has activity with many glucosinolates. Panels A-H show LC-MS extracted MRM chromatograms of desulfated glucosinolates produced *in vitro* by BtGSS after 120 min incubation with a mixture of intact glucosinolates from *Arabidopsis thaliana* Col-0. The early eluting peaks in each chromatogram indicate genuine desulfoglucosinolates formed during the enzyme assay, while the later eluting peaks show desulfoglucosinolates formed from intact glucosinolates via in-source fragmentation during MS analysis and so actually indicate the presence of intact sulfated glucosinolates used as substrates. (“ds-“ = desulfated glucosinolates; 3msop = 3-methylsulfinylpropyl; 4msob = 4-methylsulfinylbutyl; 4mtb = 4-methylthiobutyl; 5msop = 5-methylsulfinylpentyl; 7msoh = 7-methylsulfinylheptyl; 8msoo = 8-methylsulfinyloctyl; i3m = indol-3-ylmethyl; 4moi3m = 4-methoxyindol-3-ylmethyl)

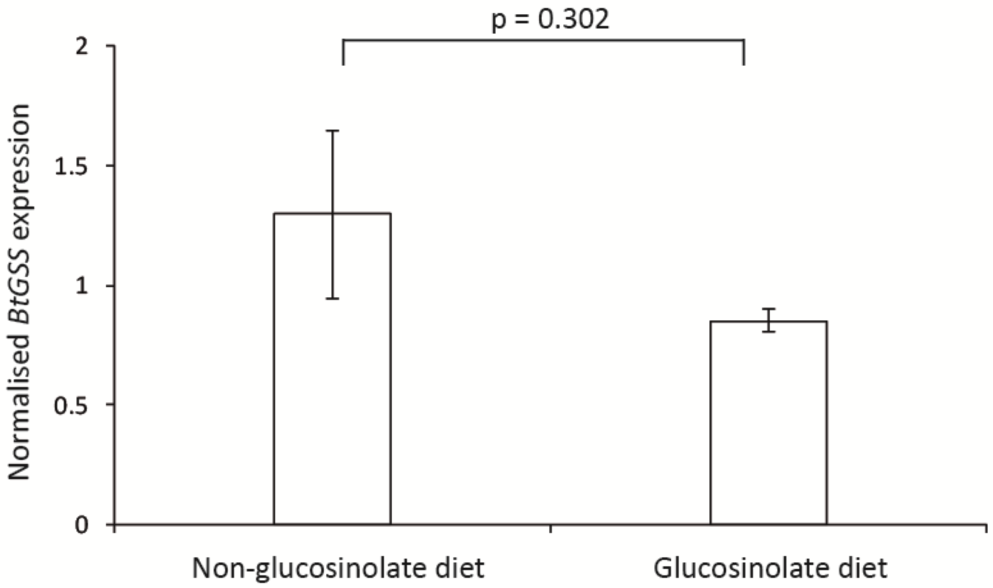

Figure Suppl. 2: Comparison of expression levels of *BtGSS* (relative to *rpl-13*) in *B. tabaci* adult insects that consumed a non-glucosinolate diet (eggplant) and those which fed on a glucosinolate-containing diet (Brussels sprouts). Results show means ± s.d., N = 3, statistical significance was examined by t-test.

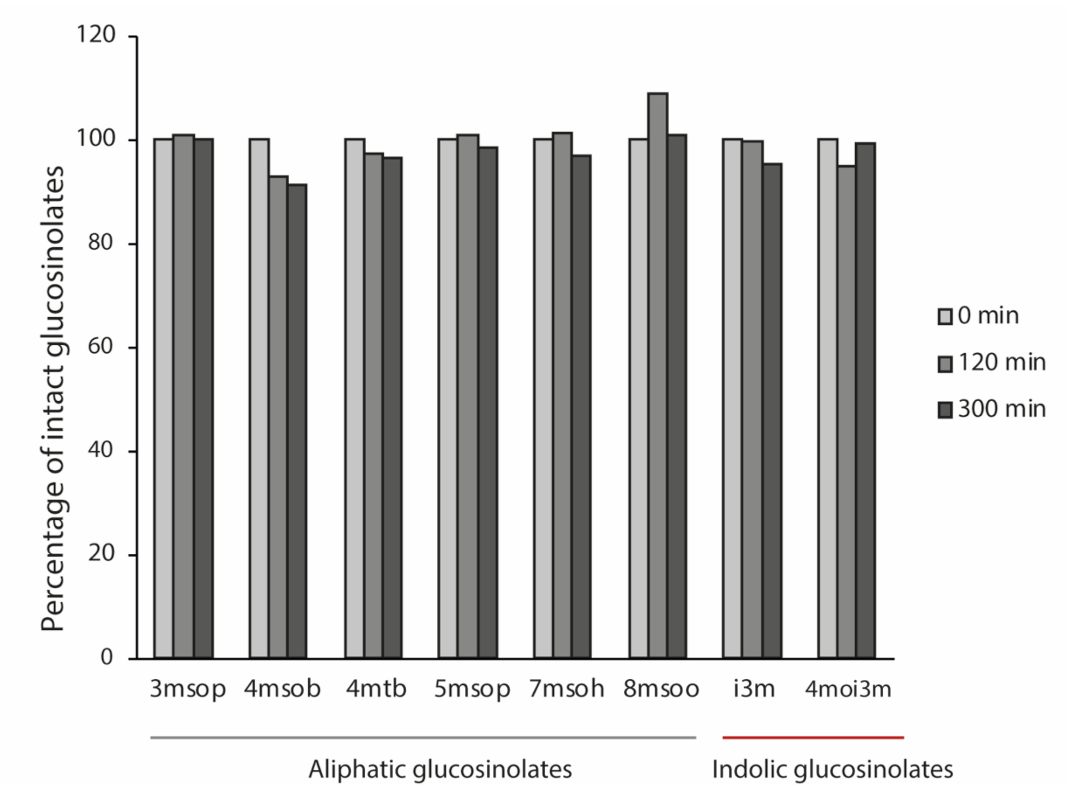

Figure Suppl. 3: Stability of glucosinolates extracted from *A. thaliana* Col-0 leaves to desulfation in aqueous solution. No spontaneous degradation to desulfoglucosinolates was observed under assay conditions even after extended incubation without sulfatase. (3msop = 3-methylsulfinylpropyl; 4msob = 4-methylsulfinylbutyl; 4mtb = 4-methylthiobutyl; 5msop = 5-methylsulfinylpentyl; 7msoh = 7-methylsulfinylheptyl; 8msoo = 8-methylsulfinyloctyl; i3m = indol-3-ylmethyl; 4moi3m = 4-methoxyindol-3-ylmethyl)

Table Suppl. 1: Primers used for RT-qPCR analysis of *BtGSS* and housekeeping gene from *B. tabaci*.

**Gene**	**Name**	**Sequence**
Bta14666 (BtGSS)	14666-qPCR-p3-FP	5'-GATCCCTGCGAGTTCCACAA-3'
	14666-qPCR-p3-RP	5'-CTTGCGATTCGACCAACTGC-3'
rpl-13	rpl-FP	5'-CATTCCACTACAGAGCTCCA-3'
	rpl-RP	5'-TTTCAGGTTTCGGATGGCTT-3'

Table Suppl. 2: Primers used for amplification of RNAi templates for *in vitro* transcription and respective products.

**Gene**	**Name**	**Sequence** (with T7 promoter sequence: TAATACGACTCACTATAGGG)
BtGSS RNAi	14666 RNAi(T7) FP	5'- CGAGAAATTAGGTGATTGGG -3'
	14666 RNAi(T7) RP	5'- GTTGGAGGCGGCTATTCT -3'
BtRNase2 RNAi	RNase2 RNAi(T7) FP	5'- CAGTGGCAAATCATCAATGCG -3'
	RNase2 RNAi(T7) RP	5'- ATGTGGAGATTTATTTACAGCCAG -3'
Scrambled BtGSS RNAi	Scrambled RNAi(T7) FP	5'- GAGAATGCCCAGAAATC -3'
	Scrambled RNAi(T7) RP	5'- CTTTAACACCGTTTCTTTC-3'
BtGSS RNAi amplicon	CGAGAAATTAGGTGATTGGGCTATCATTGTGGATGAATGGAAACTAGTAAAAGTAAATATCACCCACCTCGTGAATTTCACGCAGCTCTATTACGGCGAGACAGGACGAAACGGTGAGGAGTATTCAATGGAACTCGTCACAAACAGTCGAGTGTCCAAAATCCTTAGACGGAATCGACTCCAAGGTGATCTTCGGGAGGAATACGCGGAGCGCCGAGCCAGCTTAGAGATGACGTCATCGTGCGCGGAGAGAATAGCCGCCTCCAAC	
Scrambled BtGSS RNAi amplicon (synthetic)	AGAGAATGCCCAGAAATCGTAGGGGTGAGCACCGTCGTACCAGCTCCCTAGGAACCAGGAATACCAAGTGGCTATTACGCCGCTAGCGTGTTGTATGGGTTCGATAGTAAGTTCCATGCGGGGTCGCCCAGTGAATGAGCTAATGCGCAAAGTCGAAAAGAGTGGACAAAAGTACTACCCCTGATCACTTACGCCATAGCTGGTAGTATCCGCAAAACAGTGTATCGGATAGGATACGAAACGCTACCTGAAAGAAACGGTGTTAAAG	
BtRNase2 RNAi amplicon	CAGTGGCAAATCATCAATGCGGGCCATTGGCTGGCACTGGAACGATACCTGAGGAAGTTTGCAGAGCAGACTGGAGAGGACCTACACATCATGACTGGAATAGTGGGTGTGCTTTCTTTTGAATCTGATTCAGGGGAAAATGTGGAGATTTATTTACAGCCAG	

Table Suppl. 3: LC-MS parameters used for the multiple reaction monitoring (MRM) analyses of intact glucosinolates (negative ionization mode, API3200). CE: collision energy; CEP: collision cell entrance potential; CXP: collision cell exit potential; DP: declustering potential; EP: entrance potential; 3msop = 3-methylsulfinylpropyl; 4msob = 4-methylsulfinylbutyl; 4mtb = 4-methylthiobutyl; 5msop = 5-methylsulfinylpentyl; 7msoh = 7-methylsulfinylheptyl; 8msoo = 8-methylsulfinyloctyl; i3m = indol-3-ylmethyl; 4moi3m = 4-methoxyindol-3-ylmethyl.

Analyte	Q1 (m/z)	Q3 (m/z)	time (min)	DP (V)	EP (V)	CEP (V)	CE (V)	CXP (V)
3msop	421.8	95.9	7.6	-65	-4.5	-18	-60	0
4msob	435.9	95.8	9.5	-65	-5	-16	-60	0
5msop	449.9	95.8	10.5	-65	-5	-16	-60	0
7msoh	477.9	95.8	12.5	-65	-5	-16	-60	0
8msoo	492.1	95.8	13.6	-75	-4.5	-24	-58	0
4mtb	419.9	95.9	14.1	-60	-11	-16	-58	0
i3m	447	95.8	15.8	-65	-12	-18	-50	0
4moi3m	477.1	95.8	16.3	-65	-12	-18	-50	0

Table Suppl. 4: LC-MS parameters used for the multiple reaction monitoring (MRM) analyses of desulfated glucosinolates (positive ionization mode, API3200). CE: collision energy; CEP: collision cell entrance potential; CXP: collision cell exit potential; DP: declustering potential; EP: entrance potential; 4msob = 4-methylsulfinylbutyl; 3msop = 3-methylsulfinylpropyl; 4mtb = 4-methylthiobutyl; 5msop = 5-methylsulfinylpentyl; 7msoh = 7-methylsulfinylheptyl; 8msoo = 8-methylsulfinyloctyl; i3m = indol-3-ylmethyl; 4moi3m = 4-methoxyindol-3-ylmethyl; pOHBenz = p-hydroxybenzyl

**Analyte**	**Q1 (m/z)**	**Q3 (m/z)**	**time (min)**	**DP (V)**	**EP (V)**	**CEP (V)**	**CE (V)**	**CXP (V)**
dsAllyl	280	118	9	30	5	15	15	5
ds-3msop	344	182	7	30	5	15	15	5
ds-4msob	358	196	8.5	30	5	15	15	5
ds-5msop	372	210	9.5	30	5	15	15	5
ds-7msoh	400	238	11.5	30	5	15	15	5
ds-8msoo	414	252	12	30	5	15	15	5
ds-4mtb	342	180	12.5	30	5	15	15	5
ds-i3m	369	207	13	30	5	15	15	5
ds-4moi3m	399.1	237	13.5	30	5	15	15	5
ds-pOHBenz	346	184	10.5	30	5	15	15	5

Table Suppl. 5: LC-MS parameters used for the multiple reaction monitoring (MRM) analyses of desulfated and intact glucosinolates after *BtGSS* silencing (API6500). CE: collision energy; CXP: collision cell exit potential; DP: declustering potential; EP: entrance potential; 4msob = 4-methylsulfinylbutyl glucosinolate; ds4msob = desulfo 4-methylsulfinylbutyl glucosinolate.

**Analyte**	**Q1 (m/z)**	**Q3 (m/z)**	**time (min)**	**DP (V)**	**EP (V)**	**CE (V)**	**CXP (V)**
4msob	-436	95.8	10	-50	-5	-60	-10
ds-4msob	+358	196	8.5	30	5	15	15
